# Influencing factors of family resilience in stroke patients and family caregivers: a systematic review and meta-analysis

**DOI:** 10.3389/fpubh.2025.1716213

**Published:** 2025-12-02

**Authors:** Jingran Yang, Fang Ma, Zhisong Chen, Yu Wang, Min Zhou, Yimei Zhang, Yangjuan Bai, Qiulan Hu

**Affiliations:** 1 Department of Nursing, The First Affiliated Hospital of Kunming Medical University, Kunming, China; 2 The Second Department of Cardiology, The First Affiliated Hospital of Kunming Medical University, Kunming, China; 3 Department of Cardiology, The First Affiliated Hospital of Kunming Medical University, Kunming, China; 4 Department of Intensive Care Unit in Geriatric, The First Affiliated Hospital of Kunming Medical University, Kunming, China

**Keywords:** stroke, family resilience, patients, family caregivers, influencing factors, meta-analysis

## Abstract

**Objective:**

To systematically evaluate and explore the current status and the factors influencing family resilience in stroke patients and family caregivers through meta-analysis.

**Methods:**

This meta-analysis was registered in the International Prospective Register of Systematic Reviews (PROSPERO) [CRD42024588737]. We conducted keyword search of PubMed, Cochrane Library, Web of Science, Embase, American Psychological Association (APA), CNKI, SinoMed, CINAHL, Wan Fang Database, and VIP Database up to August 2025. The process of screening literature, extracting data, and assessing literature quality were executed by two researchers by carefully reading the titles, abstracts, and entire texts. Meta-analysis was performed using Review Manager 5.4 and Stata 16.0 software after extracting relevant data.

**Results:**

Seventeen papers were finally included, with a cumulative total of 4,252 people surveyed. The results indicated that family caregivers had a higher level of family resilience than stroke patients. Additionally, no statistically significant relationship was found between family resilience and any of the examined demographic factors, including the patients’ gender, marital status, occupational status, disease duration, stroke history, and stroke type, as well as the family caregivers’ gender, religion, residence type, chronic disease status, and the presence of co-caregivers. Patients’ family function, social support, self-efficacy, and positive coping were statistically significantly related to their family resilience. Similarly, family caregivers’ family function, social support, self-efficacy, positive coping, and burden were associated with their family resilience. However, negative coping by neither patients nor caregivers had a significant effect on family resilience.

**Conclusion:**

This study explored the factors influencing family resilience from the dual perspectives of stroke patients and family caregivers. The results should be applied in clinical practice to guide interventions for improving family resilience, thereby helping stroke patients and family caregivers cope with the challenges and rehabilitation process following a stroke. However, future longitudinal research is still needed to verify the dynamic changes and mechanisms of family resilience.

**Systematic review registration:**

https://www.crd.york.ac.uk/prospero/display_record.php?ID=CRD42024588737, identifier (CRD42024588737).

## Introduction

Stroke is an acute cerebrovascular disease caused by sudden rupture of cerebral blood vessels or damage to brain tissue due to blood vessel obstruction, which may directly lead to death (1, 2). Present data show that stroke has become the major cause of death and disability among residents worldwide (3), and the number of global stroke deaths in 2021 was approximately 7.3 million, accounting for 10.7% of all deaths (4). With advances in the treatment such as thrombolysis and improvements in acute rehabilitation services, the long-term survival rate of stroke patients is increasing, leading to a large number of stroke patients worldwide (5).

Stroke patients may experience sequelae and complications such as limb paralysis, communication impairments, and decline in cognitive function, which also inclines them towards experiencing depression, anxiety, and other kinds of negative emotional states (6). Consequently, this can result in a reduction of self-esteem and self-confidence levels, significantly influencing their physical and mental health as well as the quality of their lives. In addition, stroke patients have varying degrees of movement, swallowing, communication, and other functional impairments that limit their activities of daily living (7, 8), thus requiring lifetime care from their family caregivers (9).

Family caregivers are members of the patients’ family who help the patients carry out their daily tasks, such as eating and mobility, provide psychological, emotional, and social support, and communicate with the healthcare team regarding changes in the patient’s condition and medication adjustments (10). Studies showed that family caregivers of stroke patients bore a range of objective (quality of financial and assistance provided for activities of daily living) and subjective (feelings and perceptions associated with caregiving) care burdens (11), leading to negative psychosocial consequences for the primary caregivers and a decline in their quality of life (12). It has been confirmed that around 35% of family caregivers for stroke patients worldwide exhibit signs of anxiety and sadness (13). Negative psychological reactions of family caregivers in turn affect the rehabilitation outcomes of stroke patients (14). All above suggest that stroke is a huge challenge for the whole family and long-term care challenges not only influence the physical and emotional health of patients but also cause significant stress and a heavy economic burden for family caregivers, causing tremendous changes in family structure and functioning (15), and negatively impacting the health and well-being of the entire family (16).

However, not all families with stroke patients are negatively affected by the disease and present maladjustment. Some families adapt well to this challenging situation and draw strength from the crisis well enough to get through it (17). The study has found that some stroke families experience positive changes (18, 19). Both stroke patients and their family caregivers may experience positive psychological changes and actively utilize resources within and outside the family to get through the crisis (20). With the development of positive psychology, family researchers have paid more attention to the exploitation of family resources and strengths (21), such as family resilience, which refers to the ability and process of the family as a whole to use resources in the individual, family, and community environment to support the family in facing pressure, adversity, and crisis, and promote the family to achieve good adaptation (22, 23). Studies have shown that family resilience can facilitate families coping with the challenges posed by disease and reducing the burden on family caregivers (22, 24). And it has the potential to encourage family members to engage actively with the external environment, enabling them to obtain support and resources (25).

The global burden of stroke on patients and their family caregivers is universal, yet research into family resilience among stroke patients and family caregivers has been predominantly conducted in Asia, with very few studies from other regions. Current studies (26, 27) on the status of family resilience in stroke families have limitations such as small sample size, different sample characteristics, and diverse screening tools, etc., leading to the heterogeneity of family resilience levels in stroke families across studies. In addition, various studies reported the influencing factors related to family resilience in stroke patients and their family caregivers, including gender, age, marital status, caregiver burden, family function, social support, positive and negative characteristics of stroke patients and family caregivers, and others (23, 27–29). However, some of these studies have differing views and conclusions. A previous study reported that age of stroke patients was identified as an influencing factor of family resilience (27), but in another study the effect of age on family resilience in stroke patients was not found (30). Furthermore, some studies have proved that types of stroke have an impact on family resilience (26, 31, 32), while other studies have suggested that family resilience is not affected by types of stroke (27, 33). Coping styles of stroke patients are another factor that show controversial effects on family resilience. Some researchers claimed that there was a negative correlation between the coping styles and family resilience (27), while others concluded that there were positive correlations between them (34). The influencing factors of family resilience reported in each study were not exactly the same, and there was a lack of systematic comprehensive analysis. Therefore, the objective of this study was to conduct a systematic review and meta-analysis to summarize the current status of family resilience in stroke patients and family caregivers. Additionally, we aim to analyze the influencing factors associated with family resilience which will provide scientific basis for formulating family-centered intervention measures to improve the family resilience of stroke families, to improve the quality of life of stroke patients and reduce the burden on families and society.

## Methods

### Design

This meta-analysis was completed in accordance with the Preferred Reporting Items for Systematic Reviews and Meta-Analyses (PRISMA) statement and was registered in the International Prospective Register of Systematic Reviews (PROSPERO) [CRD42024588737].

### Literature search

The electronic databases PubMed, Cochrane Library, Web of Science, Embase, American Psychological Association (APA), CNKI, SinoMed, CINAHL, Wan Fang Database, and VIP Database were searched for eligible literature up to August, 2025. The search terms were developed using a combination of free terms and MeSH terms. A manual search of reference lists of included studies was also conducted to find additional eligible studies. The search strategy was based on synonyms of the primary search terms: “stroke” AND “family resilience.” By using PubMed as an exemplar, the strategy employed for searching relevant literature in this research is depicted in Table 1.

**Table 1:** Searching string.

Steps	Searching string
#1	Search: (“stroke”[MeSH Terms]) OR (“stroke”[Title/Abstract]) OR (“Strokes”[Title/Abstract]) OR (“Cerebrovascular Accident”[Title/Abstract]) OR (“Cerebral Stroke”[Title/Abstract]) OR (“Stroke, Cerebral”[Title/Abstract]) OR (“Cerebrovascular Apoplexy”[Title/Abstract]) OR (“Apoplexy, Cerebrovascular”[Title/Abstract]) OR (“Vascular Accident, Brain”[Title/Abstract]) OR (“Brain Vascular Accident”[Title/Abstract]) OR (“Vascular Accidents, Brain”[Title/Abstract]) OR (“Cerebrovascular Stroke”[Title/Abstract]) OR (“Stroke, Cerebrovascular”[Title/Abstract]) OR (“Apoplexy”[Title/Abstract]) OR (“CVA (Cerebrovascular Accident)”[Title/Abstract])
#2	Search: (“family resilience” [Title/Abstract]) OR (“family resilienc*” [Title/Abstract]) OR (“family resiliency” [Title/Abstract]) OR (“resilience of famil*” [Title/Abstract]) OR (“resiliency of famil*” [Title/Abstract]) OR (“family strength*” [Title/Abstract]) OR (“family hardiness” [Title/Abstract]) OR (“family adaptation” [Title/Abstract])
#3	#1 AND #2

### Inclusion and exclusion criteria

The inclusion criteria were as follows: (a) the study population was stroke patients or their family caregivers; (b) family resilience was measured or reported as a variable; (c) family resilience-related influencing factors were reported; (d) study types: cross-sectional studies, case–control studies, or cohort studies; (e) Research works clearly presented correlation coefficients or raw data that could be transformed (such as means and standard deviations). Exclusion criteria included: (a) the language used for publication was neither English nor Chinese; (b) incomplete or inaccessible data.

### Literature screening and quality assessment

All retrieved articles were initially imported into Endnote software to remove duplicates automatically. Then, two reviewers separately examined the titles and abstracts and studies that did not meet the requirements were excluded right away. Subsequently, the reviewers read the full texts of the screened materials to determine their inclusion in the ultimate analysis. Any differences of opinion were resolved by seeking advice from a third reviewer.

The same two reviewers independently appraised the quality of the eligible articles, applying the evaluation guidelines set by the Agency for Healthcare Research and Quality (AHRQ) and the Newcastle-Ottawa Scale (NOS). The criteria recommended by AHRQ for cross-sectional study included eleven items (35). For each item, if the answer was “Yes,” 1 point was awarded; if it was “No” or “Unclear,” 0 points were given. The sum of scores was an indicator of the literature’s quality, where a higher total score meant better quality. Literature with a score of 3 or below was classified as low quality, that with a score from 4 to 7 was of moderate quality, and that with a score of 8 or above was of high quality (36). NOS is equipped with eight items, specifically designed for use in cohort and case–control studies. These items are divided into three distinct dimensions: selection, comparability, and either outcome (in the context of cohort studies) or exposure (for case–control studies). In terms of quality classification, a score of 5 or less is designated as low quality, a score ranging from 6 to 7 is considered moderate quality, and a score of 8 to 9 is classified as high quality (37). If there was uncertainty regarding the quality, the article would be examined by a third reviewer.

### Data extraction

Two reviewers independently performed data extraction, and any disagreements regarding data extraction were resolved through discussions with a third reviewer. Literature data were extracted and organized using a pre-designed data extraction form, which was developed to collect basic information from the included studies. The following parameters were recorded: (a) first author; (b) year of publication; (c) country; (d) study design; (e) study population and sample size; (f) measurement tools; (g) influencing factors; (h) literature quality assessment scores.

### Data synthesis and meta-analyses

Review Manager 5.4 and Stata 16.0 were used to conduct the meta-analysis. The effect size (ES) of continuous data were described using the standardized mean difference (SMD) and its 95% confidence interval (CI), and the combined effect sizes and their corresponding 95% CIs were statistically analyzed via Z-tests. When conducting the meta-analysis of influencing factors, the Pearson correlation coefficient (r) was used as the index for measuring the effect size. The rs values from Spearman correlation analysis were transformed into *r* values (38). After utilizing Fisher’s Z transformation of the extracted data (39), we calculated the combined summary Fisher’s Z value, which was converted into a Summary *r* value (39). Then the Q-test and I^2^ statistics were applied to evaluate study heterogeneity. If *p* ≥ 0.10 and I^2^ ≤ 50%, it indicates that the heterogeneity between studies is not evident and the fixed effect model is used; if *p* < 0.10 and I^2^ > 50%, it shows that the heterogeneity is significant and the random effects model is chosen for meta-analysis (40). The Egger’s test was used to assess publication bias in the included literature, and if *p* > 0.05, it suggests that there was no publication bias. A sensitivity analysis was conducted by comparing the differences between the results of the fixed effect and random effect models, and by evaluating the impact of each study on the overall effect size using the leave-one-out method.

## Results

### Search results

**Figure 1:**
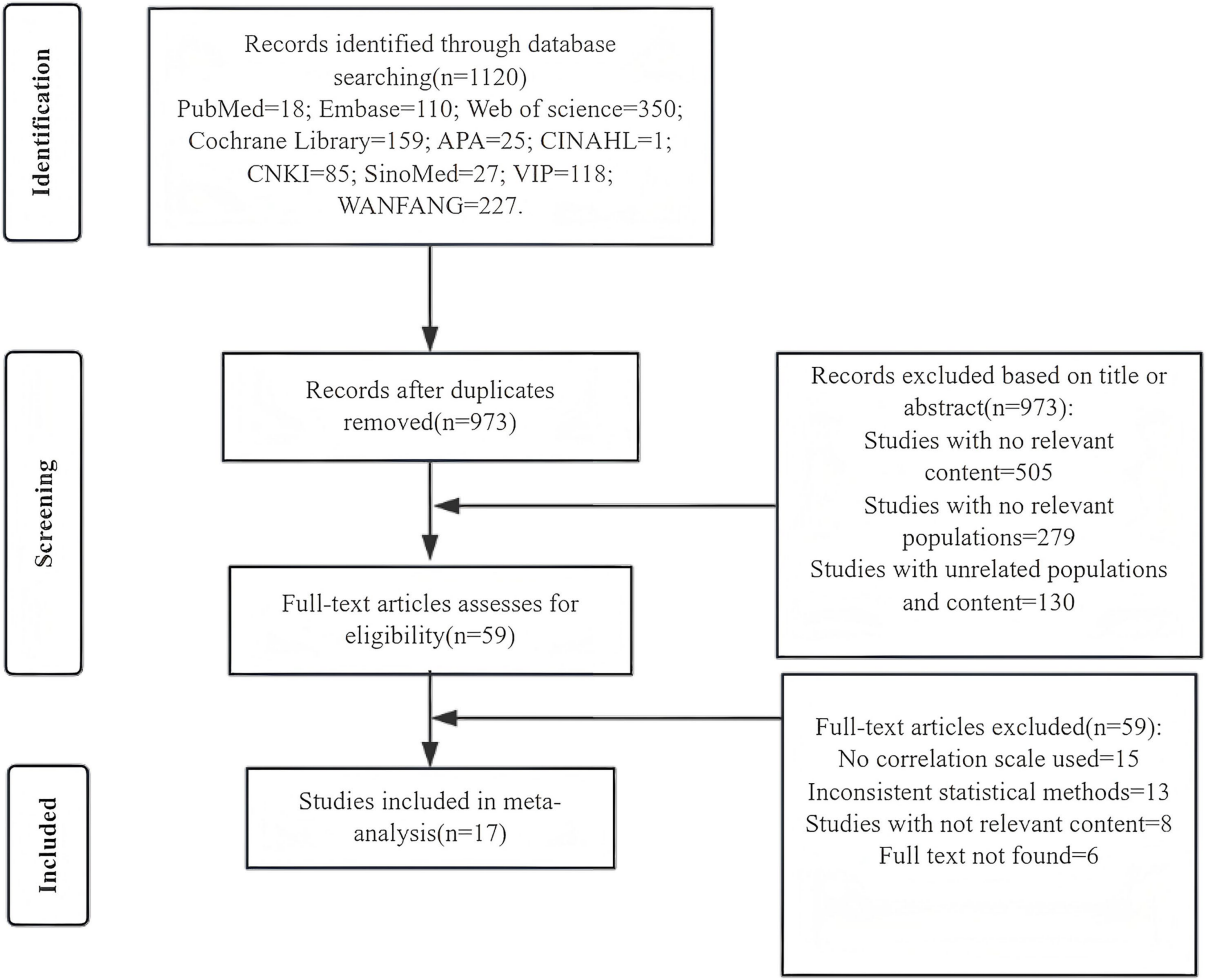
Literature screening process.

The initial search retrieved 1,120 articles from electronic databases: PubMed (*n* = 18), Embase (*n* = 110), Web of science (*n* = 350), Cochrane Library (*n* = 159), APA (*n* = 25), CINAHL (*n* = 1), CNKI (*n* = 85), SinoMed (*n* = 27), VIP (*n* = 118), WANFANG (*n* = 227), and 973 articles were left after deleting duplicates. Two reviewers initially went through the titles and abstracts, excluding those articles that were not relevant to the content of the review articles. After reading the full text of 59 articles, a total of 17 articles were included in this meta-analysis. The literature search and screening procedures are exhibited in Figure 1.

### Characteristics of the included studies

Among the 17 included studies, 7 were written in Chinese (26–28, 30, 32, 33, 41) and 10 in English (20, 23, 24, 31, 34, 42–46). All studies were published between 1996 and 2024. A total of 4,252 participants were involved, with the number of participants per study ranging from 40 to 413. Regarding measurement tools: 6 studies used the Family Resilience Rating Scale (FRRS), 7 applied the Shortened Chinese Version of the Family Resilience Assessment Scale (FRAS-C), and 4 adopted the Family Hardiness Index Scale (FHI). In terms of assessment objects: 6 studies only evaluated the family resilience of stroke patients, 8 only assessed that of family caregivers, and 3 evaluated both stroke patients and family caregivers. The characteristics of the 17 included studies are shown in Table 2.

**Table 2:** The characteristics of the included studies.

Study	Country	Study design	Sample size	Measurement tools	Population	Factors	Quality score
Man Zhang et al. (2021) (33)	China	Cross-sectional	179	FRRS	Stroke patients	1, 4, 6, 10	8
Dandan Chen et al. (2024) (27)	China	Cross-sectional	288	FRRS	Family caregivers	1, 2, 22, 23, 24, 27, 28, 29, 32, 33, 34, 36, 38	7
Xialan Ye et al. (2019) (26)	China	Cross-sectional	220	FRRS	Family caregivers	5, 7, 22, 26, 31, 32, 36, 41	7
Fen Cai et al. (2021) (30)	China	Cross-sectional	190	FRRS	Stroke patients	3, 4, 6, 9, 10, 17, 19,	6
Kunjing Han et al. (2024) (23)	China	Mixed-methods study	242	FRRS	Family caregivers	32, 35, 36, 37	6
YanQiu Lu et al. (2024) (44)	China	Cross-sectional	303	FRRS	Stroke patients	3, 4, 5, 9, 10, 11, 12, 13, 14, 27	6
Shuo Jiang et al. (2023) (28)	China	Cross-sectional	202	FRAS-C	Family caregivers	3, 4, 5, 8, 24, 25, 27, 29, 39, 40, 41	8
Mingming Ye et al. (2021) (32)	China	Cross-sectional	288	FRAS-C	Stroke patients and family caregivers	1, 7, 18, 21, 22, 24, 25, 32, 33, 34, 35, 36, 37	7
Wei Zhang et al. (2023) (34)	China	Cross-sectional	255	FRAS-C	Stroke patients	15, 16, 17, 21	8
Qian Li et al. (2024) (45)	China	Cross-sectional	350	FRAS-C	Family caregivers	32	7
Zhenfeng Zhou et al. (2024) (20)	China	Cross-sectional	253	FRAS-C	Family caregivers	32	7
Qin Ye et al. (2024) (31)	China	Mixed-methods study	379	FHI	Stroke patients	1, 3, 4, 6, 7, 9, 10, 17, 21	6
Qihang Xu et al. (2024) (24)	China	Cross-sectional	413	FRAS-C	Family caregivers	32	6
Mengfan Xu et al. (2023) (46)	China	Cross-sectional	308	FHI	Stroke patients	10, 18	8
Stevens et al. (1996) (42)	American	Cross-sectional	40	FHI	Stroke patients and family caregivers	21, 35	6
Niyomthai et al. (2003) (43)	Thailand	Cross-sectional	120	FHI	Family caregivers	42	7
Shuxian Xu et al. (2024) (41)	China	Cross-sectional	222	FRAS-C	Stroke patients and family caregivers	15, 16, 33, 34	7

For all family resilience scales, higher scores indicate higher levels of family resilience. FRRS (Family Resilience Rating Scale), consisting of 49 items, has total scores ranging from 49 to 245. FRAS-C (Shortened Chinese Version of the Family Resilience Assessment Scale), with 32 items, has total scores ranging from 32 to 128. FHI (Family Hardiness Index Scale), comprising 20 items, has total scores ranging from 0 to 60. Influencing factors: Patient:1, Age; 2, Gender; 3, Marital status; 4, Occupational status; 5, Duration of disease; 6, One or multiple episodes of stroke; 7, Types of stroke; 8, Bleeding site; 9, Education level; 10, Family monthly income; 11, Knowledge of the disease; 12, Forms of medical insurance payment; 13, Severity of illness; 14, Treatment; 15, Positive coping; 16, Negative coping; 17, Social support; 18, Family function; 19, Family adaptation; 20, Family stressors; 21, Self-efficacy. Family caregivers: 22, Age; 23, Marital status; 24, Occupational status; 25, Education level; 26, Knowledge of the disease; 27, Residence types; 28, Daily care duration; 29, Relationship with patients; 30, Co-caregivers; 31, Chronic disease; 32, Caregiver burden; 33, Positive coping; 34, Negative coping; 35, Self-efficacy; 36, Social support; 37, Family function; 38, Family support; 39, Family adaptation; 40, Family Concern Index; 41, Family monthly income; 42, Family life events.

### Literature quality evaluation results

15 studies used in this analysis were cross-sectional studies, and two were mixed-methods studies with the cross-sectional study as the quantitative part. The quality scores of the included studies ranged from 6 to 8 points, 4 of the included studies were of high quality, and the rest were of moderate quality. The evaluation results of literature quality are shown in Table 2.

### Results of the meta-analysis

#### Current status of family resilience in stroke patients and family caregivers

**Figure 2:**
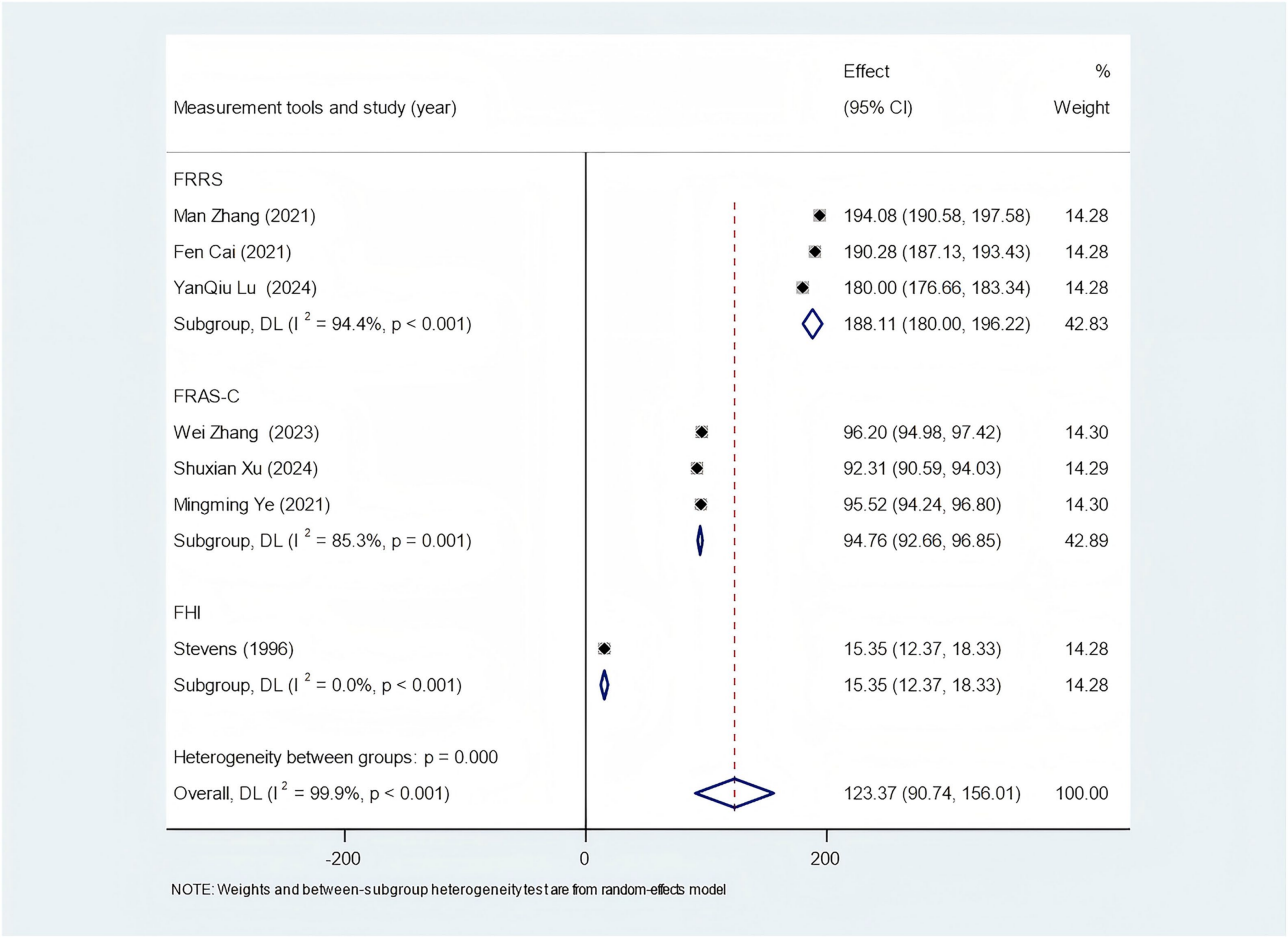
Forest plot of stroke patients’ family resilience status.

**Figure 3:**
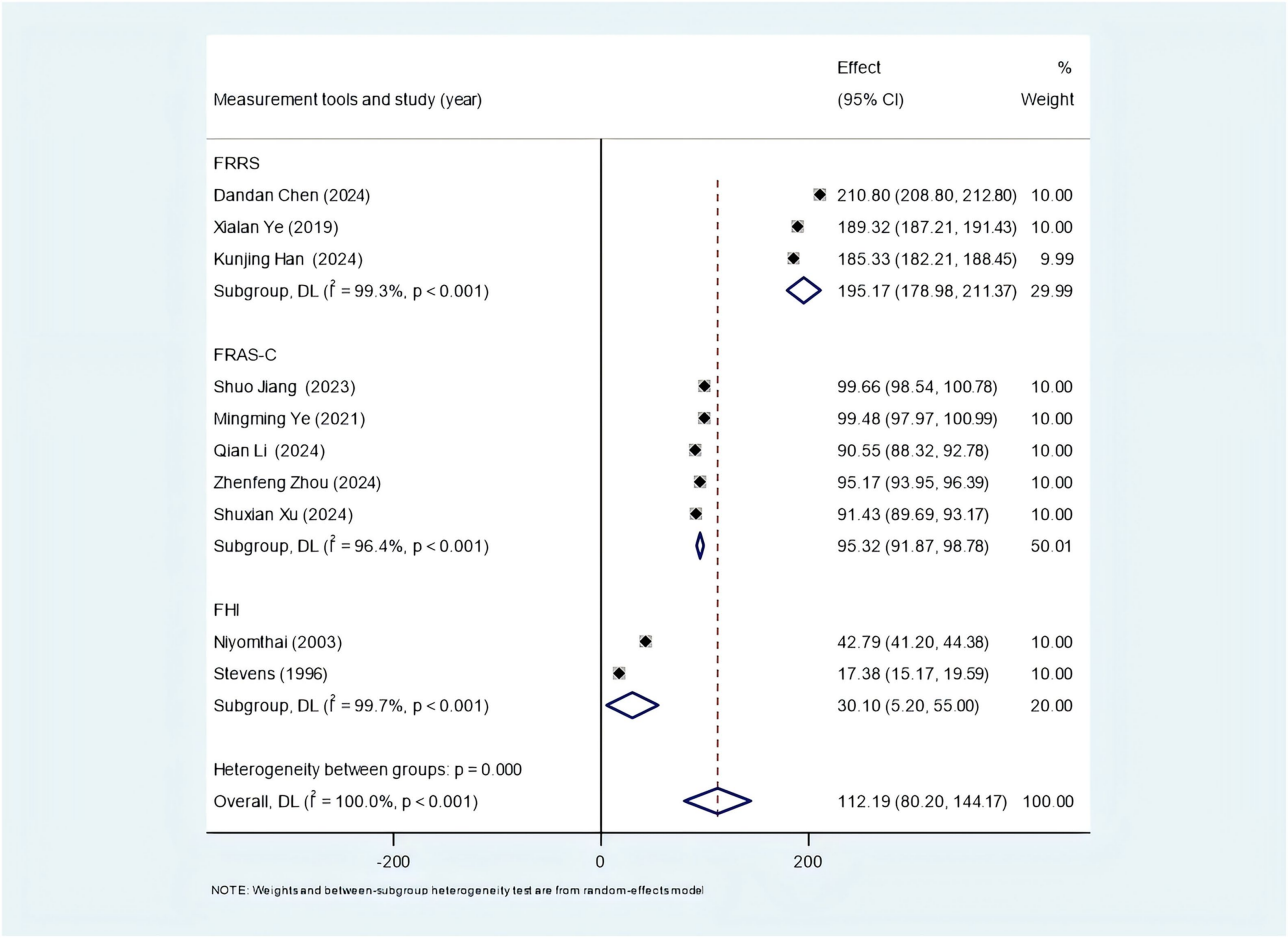
Forest plot of family caregivers’ family resilience status.

Among the 6 studies that used the FRRS scale, 3 studies measured family resilience of stroke patients, and the subgroup analysis showed a family resilience score of 188.11 (95% CI: 180.00–196.22) (as shown in the Figure 2). The remaining 3 studies measured family resilience of family caregivers, and the subgroup analysis finally showed a family resilience score of 195.17 (95% CI: 178.98–211.37) (as shown in the Figure 3). 7 studies utilized the FRAS-C. Among them, 1 study did not report the value of family resilience (24), 2 studies measured the family resilience of both stroke patients and family caregivers, 3 studies only measured the family resilience of family caregivers, and 1 study only reported the family resilience of stroke patients. After extracting and combining the data, the score of family resilience for stroke patients was 94.76 (95% CI: 92.66–96.85) (as shown in Figure 2), and for family caregivers, it was 95.32 (95% CI: 91.87–98.78) (as shown in Figure 3). Additionally, 4 studies employed the FHI. However, due to data reporting issues, only partial valid data could be extracted. For stroke patients, the family resilience score was 15.35 (95% CI: 12.37–18.33) (as shown in the Figure 2), and for family caregivers, the score was 30.10 (95% CI: 5.20–55.00) (as shown in the Figure 3).

#### The influencing factors of family resilience in stroke patients and family caregivers

To reduce heterogeneity, we analyzed the factors influencing family resilience from the perspectives of stroke patients and family caregivers, respectively. The results indicated that the relationship between general demographic factors (gender, marriage status, occupational status, duration of disease, whether patients had sustained one or multiple episodes of stroke, and types of stroke) of stroke patients and their family resilience was not statistically significant; family caregiver’s general demographics, including gender, religion, residence types, and presence of chronic disease, and presence of co-caregivers were not associated with their family resilience, as shown in Table 3.

**Table 3:** Results of meta-analysis of factors influencing family resilience in stroke patients and family caregivers (continuous data: Mean ± SD).

Population	Factors	Groups		No. of studies	Heterogeneity test		Effect model	Meta analysis		Egger’s
					*I^2^*	*P*		SMD (95%CI)	*p*	
Patients	Gender	Male	Female	5 (26, 27, 30, 31, 33)	52%	0.08	Random	−0.13 (−0.30, 0.04)	0.14	0.993
	Marital status	With spouse	Without spouse	2 (30, 33)	83%	0.01	Random	0.33 (−0.50, 1.16)	0.43	–
	Occupational status	In-service	Non-service	3 (27, 28, 33)	89%	0.0002	Random	0.06 (−0.52, 0.64)	0.83	0.395
	Duration of disease	≤14 days	>14 days	2 (28, 33)	81%	0.02	Random	0.21 (−0.34, 0.76)	0.45	–
	One or multiple episodes of stroke	Yes	No	3 (30, 31, 33)	94%	<0.00001	Random	−0.40 (−1.16, 0.36)	0.31	0.188
	Types of stroke	Haemorrhagic	Ischemic	6 (26, 27, 30–33)	80%	0.0002	Random	0.20 (−0.10, 0.50)	0.19	0.165
Family caregivers	Gender	Male	Female	4 (20, 26, 27, 32)	0%	0.89	Fixed	0.04 (−0.09, 0.16)	0.58	0.629
	Religion	Yes	No	2 (27, 32)	0%	0.69	Fixed	0.17 (−0.11, 0.46)	0.23	–
	Residence types	Urban	Rural	3 (20, 27, 28)	93%	<0.00001	Random	0.48 (−0.13, 1.09)	0.13	0.073
	Chronic disease	Yes	No	3 (26, 27, 32)	97%	<0.00001	Random	−0.44 (−1.43, 0.55)	0.39	0.330
	Co-caregivers	Yes	No	2 (20, 28)	0%	0.37	Fixed	0.12 (−0.07, 0.31)	0.22	–

As shown in Table 4, our results revealed that patients’ family function, social support, self-efficacy, and positive coping were statistically significantly related to their family resilience. Similarly, family caregivers’ family function, social support, self-efficacy, positive coping, and burden were associated with their family resilience. However, negative coping by neither patients nor family caregivers had a significant effect on family resilience.

**Table 4:** Results of meta-analysis of factors influencing family resilience in stroke patients and family caregivers (correlation coefficient *r*).

Population	Factors	No. of studies	Heterogeneity test		Effect model	Meta analysis			Egger’s
			*I^2^*	*P*		Summary Fisher’s Z (95%CI)	*p*	Summary r (95%CI)	
Patients	Family function	2 (32, 46)	90%	0.002	Random	0.54 (0.29, 0.79)	<0.0001	0.49 (0.28, 0.66)	–
	Social support	4 (30, 32, 34, 44)	93%	<0.00001	Random	0.67 (0.47, 0.86)	<0.00001	0.58 (0.44, 0.70)	0.83
	Self-efficacy	5 (23, 31, 32, 34, 42)	54%	0.07	Random	0.46 (0.38, 0.55)	<0.00001	0.43 (0.36, 0.50)	0.10
	Positive coping	2 (41, 44)	91	0.0008	Random	0.65 (0.34, 0.95)	<0.0001	0.57 (0.33, 0.74)	–
	Negative coping	2 (41, 44)	91%	0.001	Random	−0.19 (−0.48, 0.11)	0.21	−0.19 (−0.45, 0.11)	–
Family caregivers	Family function	2 (28, 32)	90%	0.002	Random	0.66 (0.38, 0.95)	<0.00001	0.58 (0.36, 0.74)	–
	Social support	4 (23, 26, 27, 32)	86%	<0.0001	Random	0.58 (0.42, 0.75)	<0.00001	0.52 (0.40, 0.63)	0.16
	Caregiver burden	6 (20, 23, 24, 26, 27, 32)	88%	<0.00001	Random	−0.47 (−0.61, −0.33)	<0.00001	−0.44 (−0.54, −0.32)	0.9
	Self-efficacy	2 (32, 42)	0%	0.81	Fixed	0.52 (0.40, 0.63)	<0.00001	0.48 (0.38, 0.56)	–
	Positive coping	3 (27, 32, 41)	94%	<0.00001	Random	0.49 (0.19, 0.79)	0.001	0.45 (0.19, 0.66)	0.8
	Negative coping	3 (27, 32, 41)	97%	<0.00001	Random	−0.3 (−0.69, 0.09)	0.13	−0.29 (−0.60, 0.09)	0.5

#### Publication bias and sensitivity analysis

In this study, Egger’s regression analysis was carried out to assess publication bias for those influence factors with over two articles incorporated. The analysis findings demonstrated that no significant publication bias was detected for any of the factors, as all *p*-values were greater than 0.05. For the meta-analysis of the influence factors, both random effects and fixed effects models were utilized. It turned out that there were slight differences in the combined effect sizes and 95% confidence intervals between the two models. These differences did not lead to qualitative changes and exerted little influence on the overall results. A leave-one-out sensitivity analysis was employed to assess the stability of our meta-analysis. This method involved iteratively omitting one included study at a time and recomputing the combined effect size for the rest. The sensitivity analysis plot (Appendix 1) clearly illustrates that the combined effect size did not undergo substantial changes with the exclusion of any single study. This implies that the outcomes of this meta-analysis are comparatively stable and reliable.

## Discussion

The results of this systematic review and meta-analysis clearly showed that in families with stroke patients, family caregivers generally exhibited higher levels of family resilience than stroke patients. From the perspective of stroke patients, the disease causes patients’ loss of self-care ability and long-term dependence on family members for daily care (47), leading to patients’ role conversion in the family, and they want to recover soon and alleviate the burden on family caregivers, which might lead to patients’ negative emotions such as anxiety and depression, feelings of guilt towards their families, and concealment of feelings (44). Current research has found that stroke patients often attempt to conceal their true feelings, perceptions, and thoughts from others, a phenomenon referred to as self-concealment (48). This situation fails to facilitate the release of patients’ negative emotions. Moreover, it does not contribute to the maintenance and development of close relationships between patients and their family caregivers. Instead, it often leads to emotional communication gaps between them (49). All above might negatively influence family communication processes of patients, which is a key point in developing family resilience according to Walsh’s family resilience model (22).

For family caregivers, although they have also experienced the severe stressor of stroke, the diagnosis and treatment process of the disease may stimulate the formation of their family’s resilience. It might be explained by the following reasons. Firstly, with the advancement of medical technology and the improvement of the stroke rehabilitation skills, family caregivers have gradually gained confidence and hope in the rehabilitation prospects of patients. Research findings suggest that the hope index is strongly associated with the level of family resilience: The higher the hope index, the greater the degree of family resilience (50). According to Hope Theory (51), hope is the internal strength and important resource for family caregivers to cope with stress. It can prompt them to transcend the current situation, downplay the pain, and actively seek effective ways and make full use of social support to meet challenges when facing stress, thus relieving stress and facilitating the development of family resilience (52). Secondly, family caregivers have the ability to recognize the social support available within their environment. For instance, they can benefit from the informational guidance provided by healthcare practitioners and the emotional support from family. Moreover, they manage to bring family members together to broaden the social support network, functioning as a buffer against the threats of disease (53). The study indicates that a high level of social support can improve family resilience, and adequate social support from family members, friends, and the community is positively correlated with higher levels of family resilience. The emotional, practical, and informational assistance received from the social network likely provides the family with the necessary resources to cope with the challenges of having a stroke patient. Finally, familial responsibility in Chinese culture is a core Confucian value that profoundly shapes caregiving expectations and family resilience. This is guided by the “Five Cardinal Relationships” (Wulun), an ethical framework articulating the hierarchical and reciprocal obligations of ruler and minister, father and son, husband and wife, older and younger brothers, and friends (54, 55). Within this framework, the father–son relationship, centered on filial piety (xiao), and the reciprocal duties among spouses and siblings play a particularly critical role in stroke care. Filial piety requires adult children to care for their ill parents, functioning as a deep-seated moral imperative. Similarly, spouses are bound by mutual lifelong support, and siblings share caregiving responsibilities. These cultural obligations drive family caregivers to prioritize care and make personal sacrifices for the caring of patients. Ultimately, this Wulun-structured duty underpins family resilience, bolstering the family’s collective capacity to cope with adversity. In contrast, Western caregiving models often emphasize individual autonomy, where caregiving is balanced with personal goals and supported by formal services rather than being viewed as a sole familial obligation (56, 57). While emotional bonds remain significant, caregiving in Western contexts is less often framed as a moral imperative and more as a voluntary choice. Cross-cultural differences suggest that family reliance on external support and personal choice in Western settings, versus family devotion and collective responsibility in Chinese contexts, may shape family resilience through distinct pathways.

Surprisingly, the social demographic data, including marital status, age, occupational status, gender, duration of disease, etc., of patients and their caregivers were found to have no substantial impact on family resilience, which is inconsistent with research findings in other populations. In a systematic evaluation of family resilience in cancer patients, it was noted that the general demographic information of cancer patients and their family caregivers, such as work status, level of education, age, and knowledge of the disease, were important influences on family resilience (58), possibly due to differences in the population with the disease. This implies that family resilience in stroke families is not primarily determined by static demographic characteristics, but rather by dynamic internal processes. Key factors such as adaptive communication, shared beliefs, flexible role allocation are more pivotal in fostering family resilience (59). For instance, it is presumed that aging might be related with family caregivers’ decreased physical and mental capacity, and thus, weaker their family resilience; yet they can offset these limitations through intergenerational collaboration or accessing external social support. This highlights that family resilience fundamentally lies in the process of adaptation, rather than being a property predicted by demographic composition. In addition, an important consideration is that the non-significant results for demographic variables might be influenced by sample characteristics. The recruitment of participants mainly from one region led to a socio-culturally homogeneous sample, which likely constrained the statistical variability of demographic factors, potentially diminishing their observed predictive power on the outcomes.

The following influencing factors of family resilience were identified in this meta-analysis: patients’ and family caregivers’ social support, family function, self-efficacy, positive coping and caregiver burden. Among them, caregiver burden was negatively correlated with family resilience, while all other factors were positively associated with it. These results are consistent with previous findings (21, 58, 60). Numerous reports had pointed out that a higher level of social support plays a crucial role in facilitating the recovery process of stroke patients and bolstering the resilience of families (61, 62). Therefore, apart from strengthening social support on an individual level, families should have access to community support services like family therapy, support groups, and long-term care services. These services can provide a platform for stroke patients and their family caregivers to openly exchange experiences, share burdens, explore coping strategies, and ultimately improve the quality of their relationships (63, 64).

General self-efficacy reflects family caregivers’ confidence in providing care. The higher level of family caregivers’ self-efficacy, the longer they tend to persist in caring for patients and the better they can cope with various stressors caused by the patients’ illnesses (65). Higher self-efficacy in stroke patients fosters greater engagement in self-management, which directly alleviates caregiver burden and strengthens the family’s collective confidence, creating a positive cycle that enhances overall family resilience (66, 67). It is strongly recommended to conduct cognitive-behavioral adjustment training and therapeutic approaches for emotion enhancement to boost the self-efficacy of stroke patients and family caregivers, thereby achieving good family resilience (46). Individuals who confront adversity positively and adopt positive coping strategies are more likely to proactively seek medical resources, develop a deeper understanding of their health conditions, and actively seek support from relatives and friends. In turn, this process fosters positive psychological states, directs the family onto a trajectory of adaptation, and ultimately builds family resilience (68). Medical staff should focus on strengthening health education and supportive encouragement for stroke patients and family caregivers to help them develop and apply positive coping strategies, thereby promoting the development of family resilience.

This study confirms a negative correlation between family resilience and caregiver burden, a finding that can be further explained by family systems theory (69). When family caregivers bear excessive burden due to long-term physical and mental exhaustion (e.g., emotional exhaustion, physical fatigue), their individual resources tend to be depleted, which directly impairs the core functions of the family system, including the abilities for effective communication, collaborative decision-making, and emotional support. Such impairment of system functions further hinders the family’s overall capacity to adapt to adversities (22, 70). Future efforts should prioritize accessible external supports such as professional community care, practical training, and peer-support networks to reduce caregiver burden.

## Limitations

Although this study has made efforts to comprehensively analyze multiple research results, there are still inevitably some limitations. First, the heterogeneity included in the study is relatively high, mainly reflected in differences in study design, sample characteristics, measurement tools, and evaluation time points. This heterogeneity may lead to the accuracy and reliability of the research results being affected to a certain extent, and making us need to be more cautious when interpreting and generalizing the research conclusions. For example, the measurement tools for family resilience vary in different studies, covering different dimensions and focuses, which may cause deviations when comparing different research results and make it difficult to form a unified and precise conclusion. Second, although numerous factors influence the family resilience of stroke patients and family caregivers, there are far fewer common factors involved in two or more studies and we were unable to include clinical variables related to stroke due to data constraints. This may lead to bias in the meta-analysis results. Third, as cultural and medical contexts vary across countries, and the majority of the included studies focused on Asian populations, caution is warranted regarding the geographic generalizability of the findings to all stroke patients. Finally, most of the included studies are observational, which limits our ability to make a definitive determination of the causal relationship between the factors and family resilience. Although we could find associations between some factors and family resilience in these studies, we could not conclusively confirm the causal relationship, which poses some difficulties in understanding the formation mechanisms of family resilience deeply. Future research needs to adopt more rigorous experimental designs, such as conducting longitudinal studies, in order to further clarify the causal relationship between these factors and provide a more solid theoretical basis for the development of targeted interventions.

## Conclusion

In conclusion, this systematic review and meta-analysis examined the current status of family resilience in stroke patients and family caregivers, as well as the influencing factors of family resilience in them. We found that in stroke families, family caregivers’ level of family resilience was higher than that of stroke patients. This finding suggests that patients should be considered as a crucial target for interventions to foster family resilience across the entire family. This study also revealed that multiple factors were recognized as having an impact on the family resilience of stroke patients and family caregivers, including social support, family function, self-efficacy, positive coping of family caregivers and stroke patients, and caregiver burden. Nevertheless, given the limitations of current research, more large-sample, high-quality longitudinal and intervention studies should be conducted in the future. Through long-term tracking and observation of families throughout disease progression, these studies can deeply analyze the dynamic of family resilience and the mechanisms of its influencing factors in stroke patients and family caregivers.

## Data Availability

The datasets used and/or analyzed during the current study are available from the corresponding author on reasonable request.
