# Supplementary material for: Influencing factors of family resilience in stroke patients and family caregivers: a systematic review and meta-analysis
Source: Front Public Health. 2025 Dec 2;13:1716213. doi: 10.3389/fpubh.2025.1716213 (PMC12705361; doi:10.3389/fpubh.2025.1716213)
Supplement: Supplementary file 1 [file Data_Sheet_1.docx]

**Leave-one-out Sensitivity Analysis Plot for Influencing Factors on Family Resilience in Stroke Patients and Family Caregivers**

1. Gender of patients

1. Occupational status of patients

1. One or multiple episodes of stroke

1. Types of stroke

1. Gender of family caregivers

1. Residence types of family caregivers

1. Chronic disease of family caregivers

1. Patients social support

1. Patients self-efficacy

1. Family caregivers social support

1. Family caregivers burden

1. Family caregivers positive coping

1. Family caregivers negative coping
